# A novel diversity method for smartphone camera-based heart rhythm signals in the presence of motion and noise artifacts

**DOI:** 10.1371/journal.pone.0218248

**Published:** 2019-06-19

**Authors:** Fatemehsadat Tabei, Rifat Zaman, Kamrul H. Foysal, Rajnish Kumar, Yeesock Kim, Jo Woon Chong

**Affiliations:** 1 Dept. of Electrical and Computer Engineering, Texas Tech University, Lubbock, TX 79401, United States of America; 2 Dept. of Civil Engineering and Construction Management, California Baptist University, Riverside, CA 92504, United States of America; Zapadoceska univerzita, CZECH REPUBLIC

## Abstract

The advent of smartphones has advanced the use of embedded sensors to acquire various physiological information. For example, smartphone camera sensors and accelerometers can provide heart rhythm signals to the subjects, while microphones can give respiratory signals. However, the acquired smartphone-based physiological signals are more vulnerable to motion and noise artifacts (MNAs) compared to using medical devices, since subjects need to hold the smartphone with proper contact to the smartphone camera and lens stably and tightly for a duration of time without any movement in the hand or finger. This results in more MNA than traditional methods, such as placing a finger inside a tightly enclosed pulse oximeter to get PPG signals, which provides stable contact between the sensor and the subject’s finger. Moreover, a smartphone lens does not block ambient light in an effective way, while pulse oximeters are designed to block the ambient light effectively. In this paper, we propose a novel diversity method for smartphone signals that reduces the effect of MNAs during heart rhythm signal detection by 1) acquiring two heterogeneous signals from a color intensity signal and a fingertip movement signal, and 2) selecting the less MNA-corrupted signal of the two signals. The proposed method has advantages in that 1) diversity gain can be obtained from the two heterogeneous signals when one signal is clean while the other signal is corrupted, and 2) acquisition of the two heterogeneous signals does not double the acquisition procedure but maintains a single acquisition procedure, since two heterogeneous signals can be obtained from a single smartphone camera recording. In our diversity method, we propose to choose the better signal based on the signal quality indices (SQIs), i.e., standard deviation of instantaneous heart rate (*STD*–*HR*), root mean square of the successive differences of peak-to-peak time intervals (*RMSSD*–*T*), and standard deviation of peak values (*STD*–*PV*). As a performance metric evaluating the proposed diversity method, the ratio of usable period is considered. Experimental results show that our diversity method increases the usable period 19.53% and 6.25% compared to the color intensity or the fingertip movement signals only, respectively.

## Introduction

Heart rhythm has been used as a significant indicator in monitoring cardiovascular healthiness e.g. cardiac arrhythmias may indicate atrial fibrillation (AF) which is correlated to the risk of stroke and heart failure [1, 2]. Slow or fast heart rate in addition to heart rhythm could also point out the heart healthiness. According to the World Health Organization (WHO), heart diseases, including heart attack, stroke, heart failure, and heart valve problems, are reported as the main causes of more than 30% of all deaths around the world [3]. Since heart diseases can be asymptomatic and intermittent, especially in early stages, detecting heart diseases has been a major challenge to clinicians. Therefore, a simple heart rhythm monitoring technique (that is readily available without requiring additional electrodes/sensors) is needed for outpatient use and daily activities [2, 4]. As smartphones prevail around the world, and smartphone cardiovascular apps are developed and used to monitor users’ health [1, 5, 6], the opportunity exists to provide the medical community with a quality smartphone cardiac monitoring technology. Video camera sensors embedded in smartphones enable acquiring heart rate (or pulse rate) from users’ fingertips. For example, time series signals, called smartphone photoplethysmogram (PPG), are obtained from color intensity changes [7] of successive fingertip images taken by a smartphone video camera. These signals provide physiological information including oxygen saturation, heart rate, and respiratory rate [8–10]. Previous studies showed that the smartphone PPG signal could be used for AF determination and discriminating AF from premature atrial contractions (PACs), premature ventricular contractions (PVCs) and normal heart rhythm [1, 5, 6, 11]. Moreover, the advent of highly sensitive image sensors in smartphone video cameras has enabled acquisition of fingertip movement caused by heart pumping [12].

The heart rate estimated from these smartphone signals is 90% accurate when they are acquired without motion. However, there are different factors that limit accurate measurements of heart rhythm and heart rate variation when using smartphones. Some of these factors are limited sampling rate of smartphones compared to clinical devices, heating problem of the flash light in long term measurement and the experimental artifacts induced during acquisition step [13–15]. Since the first two factors are related to the structure of the smartphones, filtering out the experimental artifacts [also called motion and noise artifact (MNA)] is relatively more essential to overcome than the other factors. Walking, running, hand movement, and tremor are some examples of experimental conditions that produce different experimental artifacts.

To overcome the MNA, different MNA detection/reduction approaches have been proposed: hardware-based and software-based. The hardware-based MNA detection approaches measure pure MNA signals from additional MNA-focused hardware, e.g. accelerometer [16, 17] and use them to remove/reduce MNAs. However, these approaches require additional signals together with main physiological signals. Moreover, the hardware-based approaches may cause false positive MNA detections in that the physiological signal is clean but the hardware estimates that the signal is corrupted by MNAs. Differently, from the hardware-based approaches, the software-based approaches are based on signal processing techniques/algorithms. For example, blind source separation [18–25], time- or frequency-domain parameters [26, 27], and adaptive filter techniques [28] are introduced to detect MNAs. A concept of signal quality index (SQI) is widely adopted in the MNA detection methods using time- or frequency-domain parameters since the SQI can effectively quantify the amount of MNAs in physiological signals [17, 28–33]. In addition, data fusion techniques have been introduced to reduce the effect of MNAs by exploiting the diversity from multiple sensors [34–40]. The data fusion is adopted for respiratory rate estimation from noisy signals measured with photoplethysmograph (PPG), impedance pneumograph (IP), arterial blood pressure (ABP) and peripheral arterial tonometry waveform (PAT) [36, 37]. Different modulation sources are applied to extract the respiratory rate from a single lead ECG [39]. The heart rate, signal quality indices, and data fusion approach are adopted together to reduce the effect of false alarms in the intensive care unit (ICU) [38, 40].

In this paper, we propose a novel diversity method which exploits the diversity gain to obtain reliable heart rate information, i.e. to increase the ratio of the clean usable segment to be used to calculate heart rate. We do this by selecting the better signal between the two signals (color intensity or fingertip movement) based on SQIs. As a result, the proposed method will provide more usable periods compared to the non-diversity method, e.g. the color intensity signal only. We consider two different types of heterogeneous smartphone signals obtained from a single smartphone camera recording: 1) color intensity signal [7], and 2) fingertip movement signal [12]. These two acquired signals are heterogeneous since they extract different information, i.e., the color intensity signal measures blood flow change on a fingertip while the fingertip movement signal measures the subtle movement of fingertip initiated by heart pumping. To exploit the diversity from these two heterogeneous smartphone signals, the proposed method 1) first divides the smartphone signals (color intensity and fingertip movement signals) into segments, and 2) then calculates the SQIs’ values of each segment. 3) Then, for each time slot, the proposed method selects the better segment between the two segments (color intensity and fingertip movement segments).

## Materials and methods

### Experimental procedure

**Fig 1:**
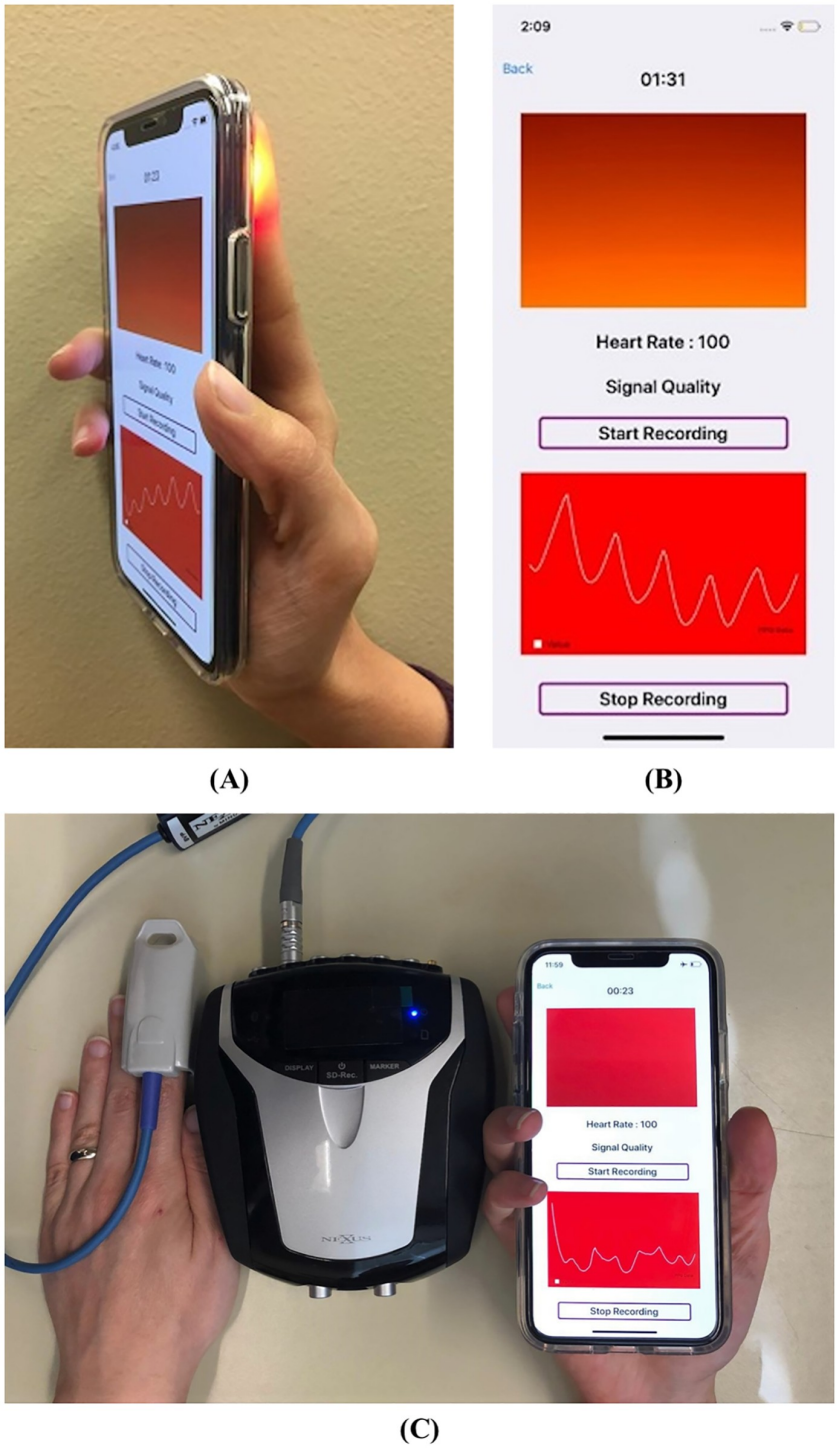
Smartphone signal acquisition procedure. (A) A subject’s fingertip is placed on a smartphone lens during the signal acquisition procedure, (B)the screenshot of our developed app displays acquired PPG signal, instantaneous heart rate, and remaining time on a smartphone screen in real-time during the measurement procedure, (C) PPG sensor of the Nexus is measuring the PPG signal from the subject’s other finger as a gold standard measurement. The hand with the NeXus PPG sensor is in a still position during the total duration of the measurement procedure.

The smartphone data and PPG data which are used in this paper are acquired under a protocol approved by the Institutional Review Board (IRB) (IRB#: IRB2016-764) at the Texas Tech University. We recruited 15 healthy subjects whose ages are in the range of 18 to 80. The recruited subjects were not diagnosed with cardiovascular problems. From the recruited subjects, smartphone signals were acquired using an iPhone X. Specifically, each subject was asked to sit on a chair in a room with ambient light and place his/her fingertip on a camera lens as shown in Fig 1A. When our developed smartphone app starts, the flashlight beside the lens is turned on automatically and the smartphone camera records images.

During the measurement procedure, our smartphone app displays the image of fingertip taken by smartphone camera as a red rectangle at the top of the screen shown in Fig 1B. These acquired images by smartphone camera are the source images for further analysis explained in detail in the subsection Signal Acquisition Step. As shown in Fig 1B the smartphone app also displays the acquired PPG and instantaneous heart rate. The total duration of the measurement procedure is 2 minutes. During the measurement procedure, the smartphone’s camera and the lens are fully covered with the subject’s fingertip (see Fig 1A); and at the same time placing his/her finger of the other hand inside the PPG clip sensor of the NeXus 10 mark-II (see Fig 1C) [41]. To induce the MNA in the smartphone signals, subjects are asked only to move the hand which holds the smartphone in a left-right or/and up-down direction. The total duration of the movements (up-down and left-right directions) is 30 seconds. During the movement phase, the smartphone’s camera and the lens are still fully covered with their fingertip, while keeping the other hand with the NeXus PPG sensor in a stable position.

**Fig 2:**
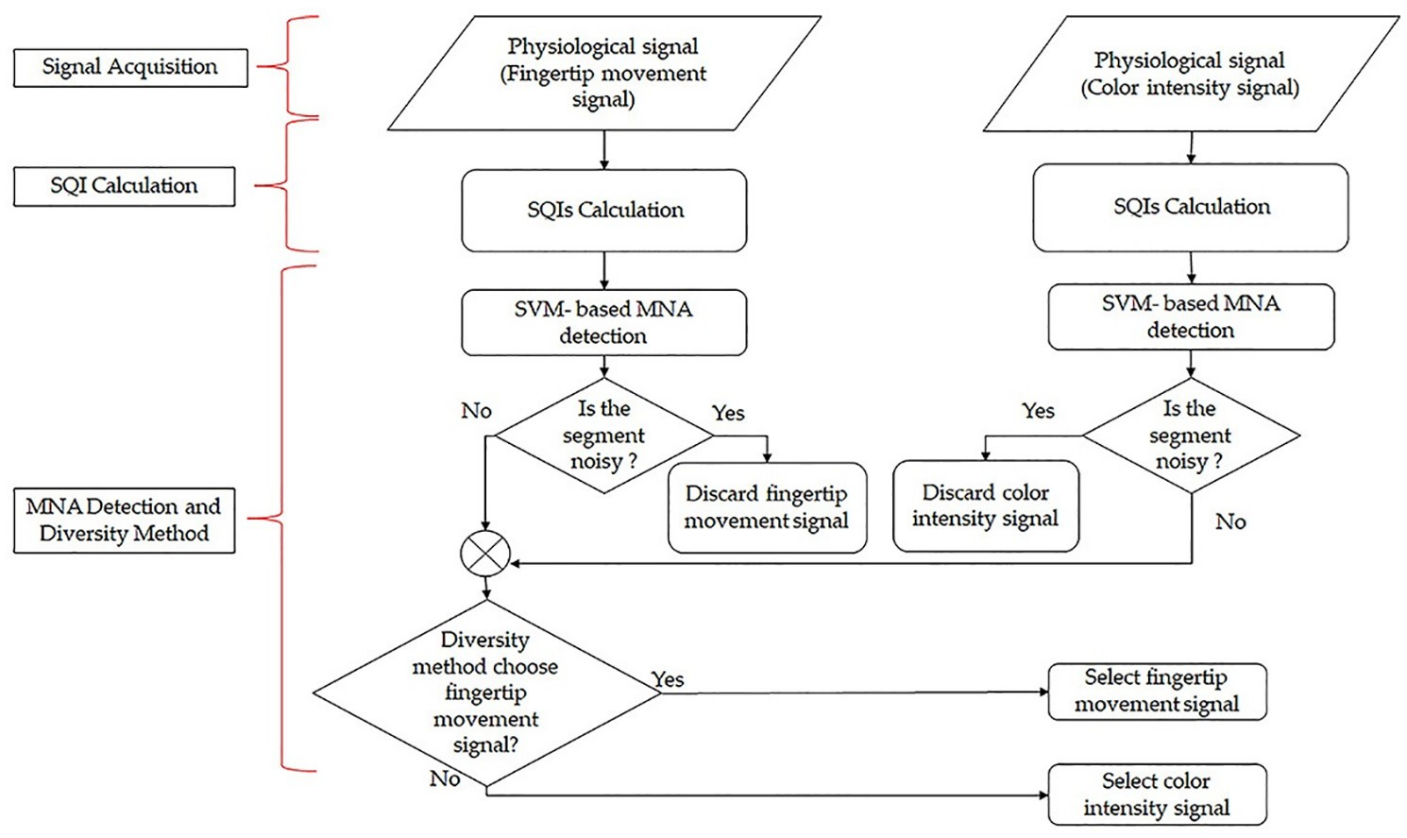
A flow chart of our proposed diversity method.

Our proposed diversity method for smartphone-based heart rhythm signals consists of 1) signal acquisition, 2) SQIs calculation, and 3) signal selection steps as shown in Fig 2. The detailed description of these three steps is presented in the following subsections.

### Signal acquisition step

**Fig 3:**
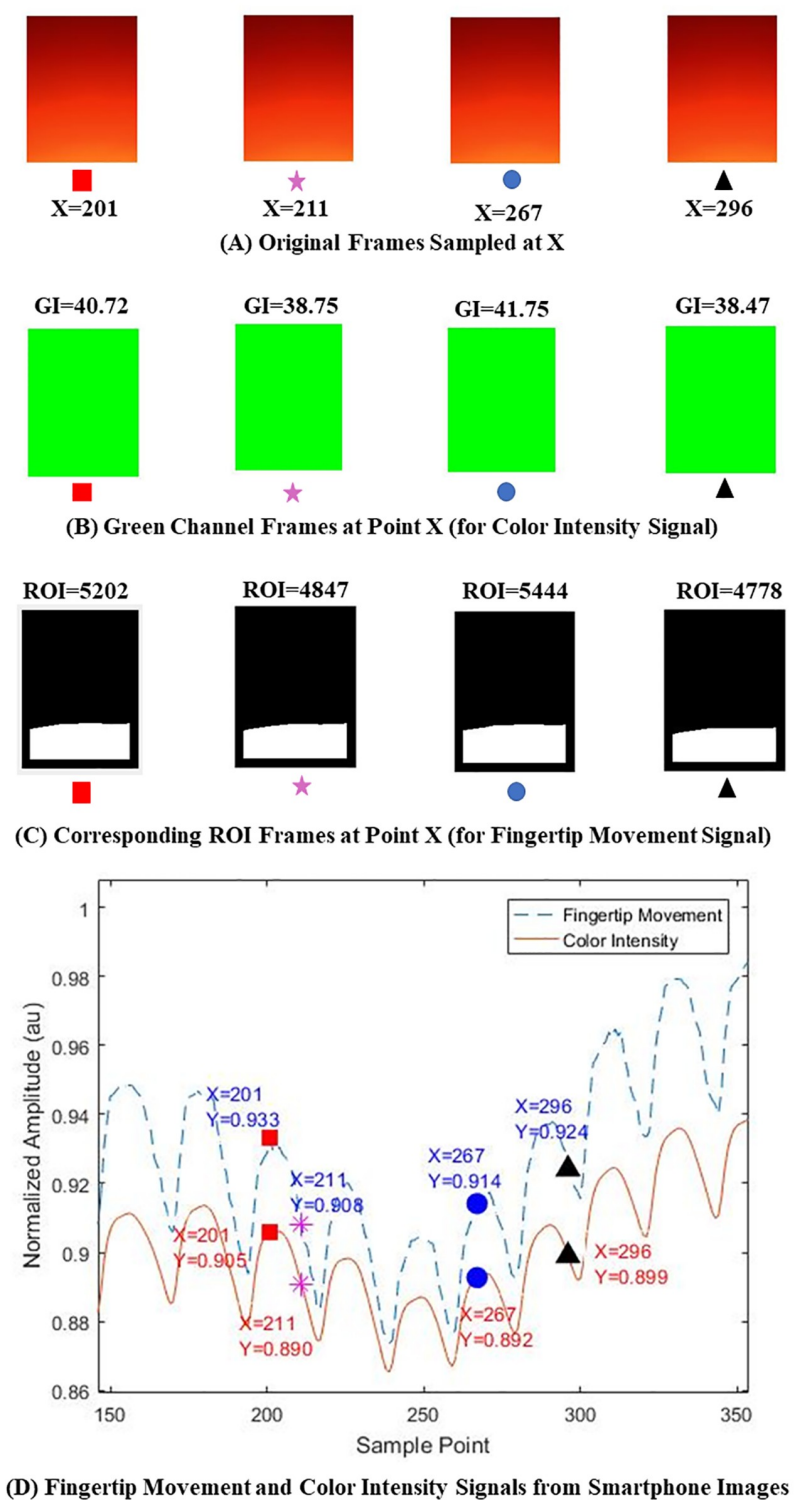
Color intensity signal and fingertip movement signal obtained from a smartphone camera video recording consisting of 3,600 successive images. (A) Four source images (201^st^, 211^th^, 267^th^, and 296^th^ image), (B) green color intensity values, visualized as a mapped image in each source image, (C) ROI in each source image, and (D) color intensity (red solid line) and fingertip movement (blue dashed line) signals. In Fig 3D, the y-values of 201^st^(red-rectangle), 211^th^(purple-star), 267^th^(blue-circle), and 296^th^(black-triangle) for color intensity signal (or red solid line) are directly calculated from the four average intensity values of green color in Fig 3B of the source images. On the other hand, the y-values of the four points on the fingertip movement signal (or blue dashed line) in Fig 3D are directly calculated from the four ROI sizes, shown in Fig 3C of the source images, shown in Fig 3A.

From a single smartphone video recording (see Subsection Experimental Procedure), two heterogeneous types of signals are acquired in the signal acquisition step: 1) color intensity signal [7], and 2) fingertip movement signal [12]. Here, the 3,600 images come from a 120-second recording time with a 30-frame per second (fps) sampling rate (30 (fps) x 120 (secs) = 3,600 frame images). Specifically, as an example, the four images, which are 201^st^ (red-rectangle), 211^th^ (purple-star), 267^th^ (blue-circle), and 296^th^ (black-triangle) images among the 3,600 images are shown in Fig 3A. The four average intensity values of green color in Fig 3B of the source images (Fig 3A) are used to calculate the color intensity signal. On the other hand, the sizes of four regions of interest (ROI) shown in Fig 3C of the source images (Fig 3A) are used to calculate the fingertip movement signal. Both signals are one-dimensional time-series signals (see Fig 3D) obtained from two-dimensional successive images (see Fig 3A). Therefore, the four images shown in Fig 3A are the sources of the four points in Fig 3D. Specifically, the y-values of the four markers on the red solid line are directly calculated from the four average intensity values of green color in Fig 3B of the source images. The y-values of the four points on the fingertip movement signal (or blue dashed line) in Fig 3D are directly calculated from the sizes of four regions of interest (ROI) shown in Fig 3C. In this example, a smartphone recording consisting of 3,600 successive images results in a one-dimensional color intensity signal and a one-dimensional fingertip movement signal, each of which is composed of 3,600 successive points. The detailed procedure of calculating green channel values from the original images is described in subsection ‘Color Intensity Signal’ while the procedure of calculating sizes of ROIs is presented in subsection ‘Fingertip Movement Signal’.

#### Color intensity signal

**Fig 4:**
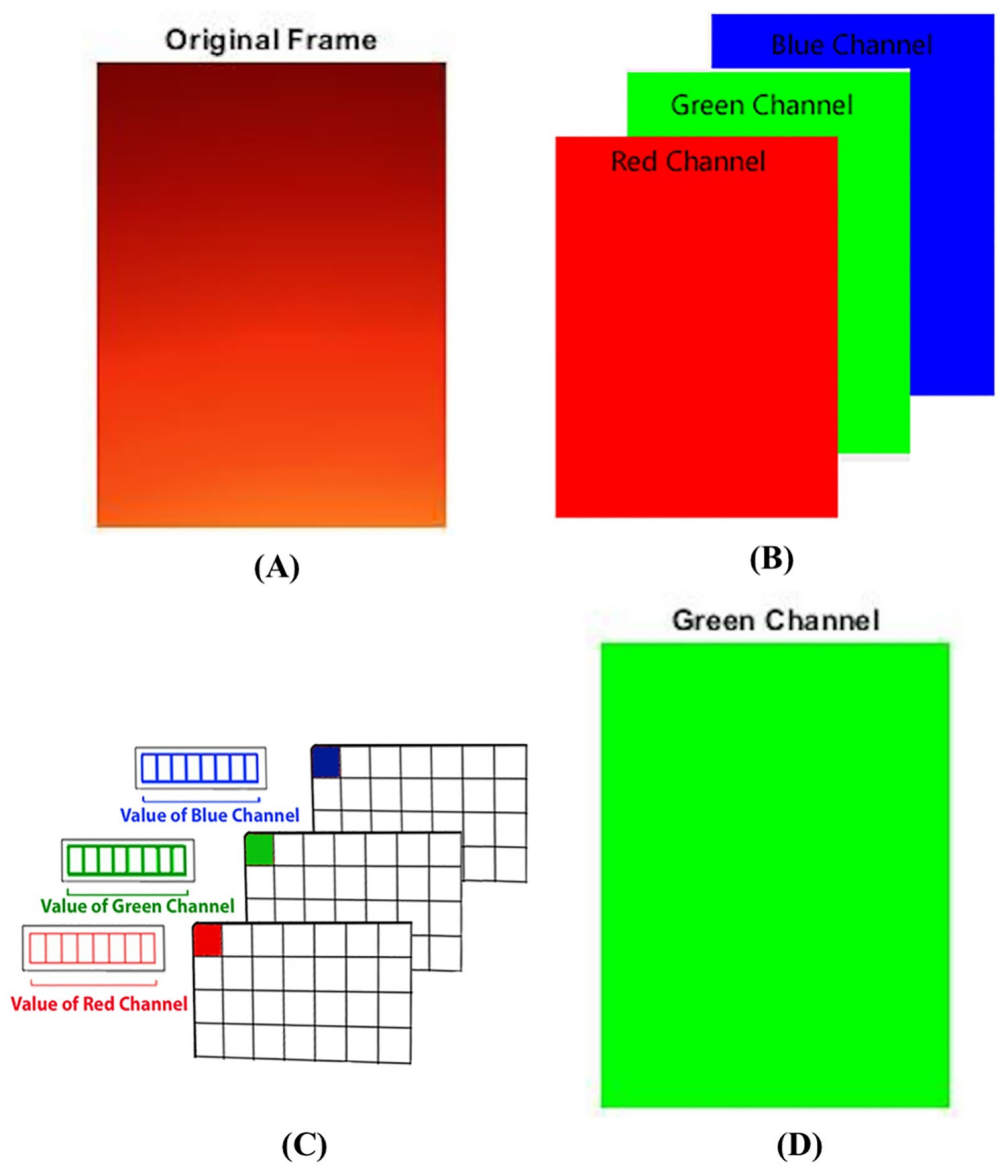
Procedure of obtaining a point value on a color intensity signal from a source image. (A) source image, (B) red, green, and blue channel images of the source image, (C) bit representation of intensity values for each color channel image and (D) green channel image consisting of the green values at each pixel of the source image.

Color intensity signal is derived from source images as follows: 1) green channel image extraction, and 2) average color intensity calculation. Fig 4 shows the procedure of getting a point on a color intensity signal from a source image. Each source image (see Fig 4A) is represented by RGB 888 image format which consists of three color channels: red (R), green (G) and blue (B) (see Fig 4B). From each pixel in a source image, the green color intensity value among R, G, and B is extracted as shown in Fig 4C. Here, green channel is chosen since the absorption of green light in the oxyhemoglobin is most sensitive among three colors [42]. As a result, a green channel image consisting of the green values at each pixel of the source image is shown in Fig 4D. The average value of these pixels on the green channel image (Fig 4D) are mapped into one point of a color intensity signal. This procedure is repeated for each successive source image.

#### Fingertip movement signal

**Fig 5:**
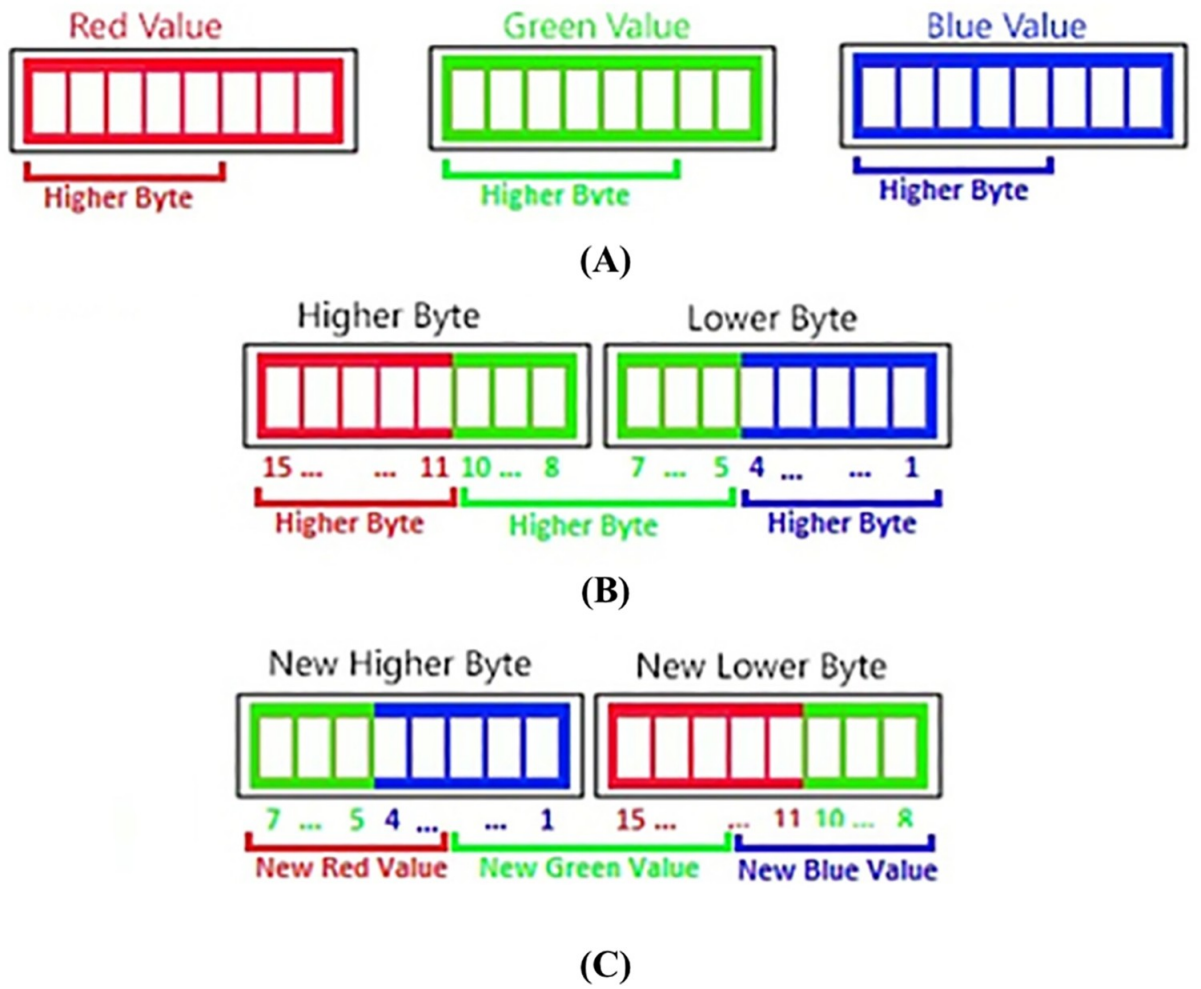
Bit rearrangement substep applied to every pixel of a source image for obtaining fingertip movement signal. (A) RGB888 format representing a source image, (B) conversion of RGB888 format into RGB 565 format, and (C) bit rearrangement.

Fingertip movement signal is obtained from source images by the following steps: 1) bit rearrangement, 2) edge detection, 3) smoothing, 4) binarization, and 5) ROI calculation. Fig 5 shows the bit rearrangement step which is applied to every pixel of a source image. Using a common method, a source image which is represented by RGB888 image format of three bytes (see Fig 5A) is reduced into RGB565 image format of two bytes (see Fig 5B), and the RGB565 image is rearranged as shown in Fig 5C.

**Fig 6:**
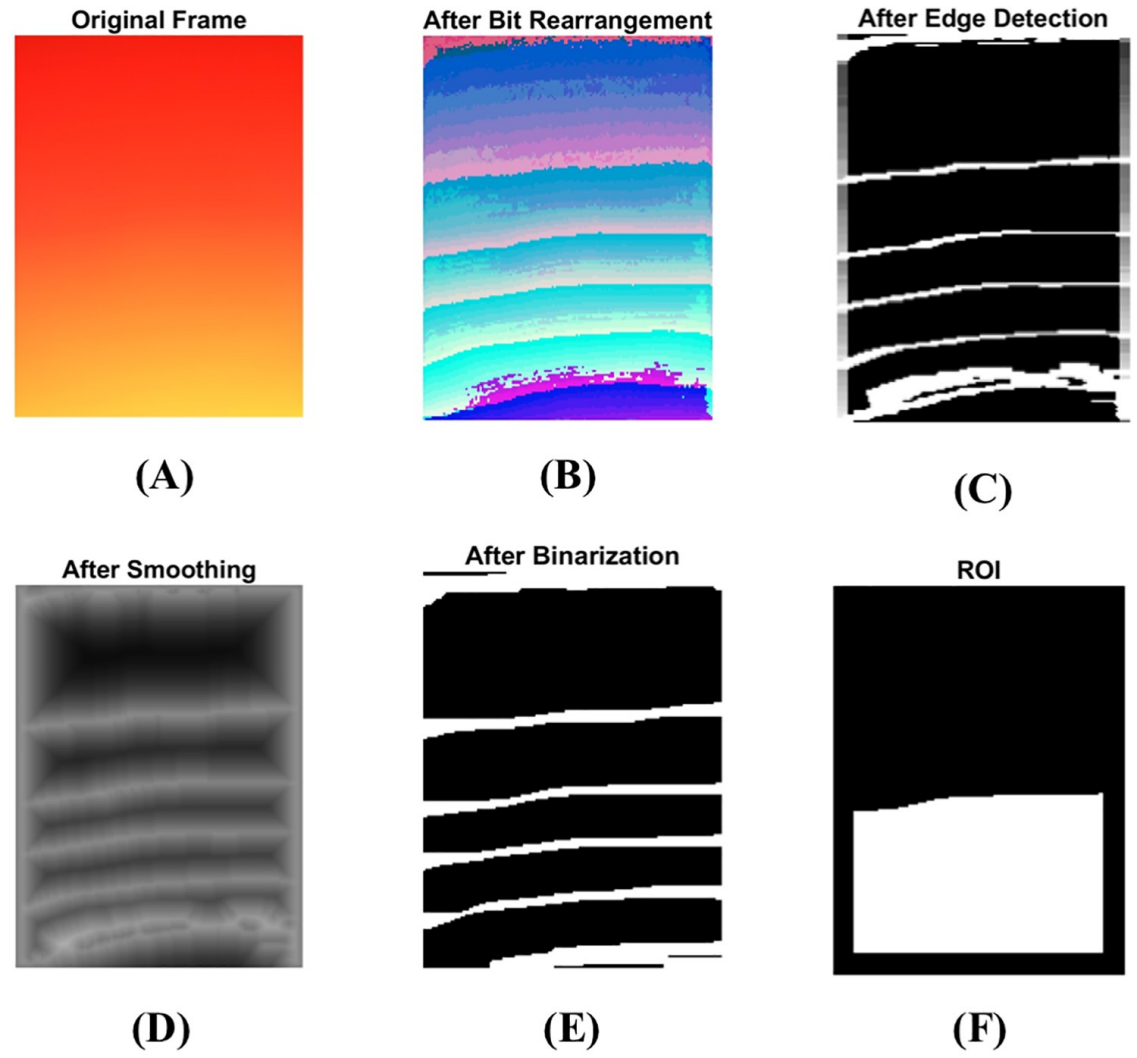
Preprocessing of smartphone video camera images before acquiring a fingertip movement signal. (A) The original frame from smartphone recording before any processing, (B) bit rearrangement, (C) edge detection, (D) smoothing, (E) binarization, and (F) ROI size which is calculated as a sample point of the fingertip movement signal.

The bit rearrangement process is applied to the original image shown in Fig 6A. After bit rearrangement, in each color, the locations of the bits near the most significant bit (MSB) are exchanged with the locations of the bits near the least significant bit (LSB). This process enhances major changes coming from MSB, making variations visually more apparent. After this bit rearrangement procedure (see Fig 6B), images are converted into grayscale images. On grayscale images, edge detection, smoothing, and binarization steps are performed sequentially. In the edge detection step, an edge is detected using the differential operator method (see Fig 6C), where edges are a set of points having larger differential value than a pre-defined threshold. Using distance transform and anisotropic diffusion [43, 44], the edges are smoothed by removing the discontinuity around the edges in the smoothing step (see Fig 6D). Then, the smoothed edges are binarized in the binarization step (see Fig 6E).

**Fig 7:**
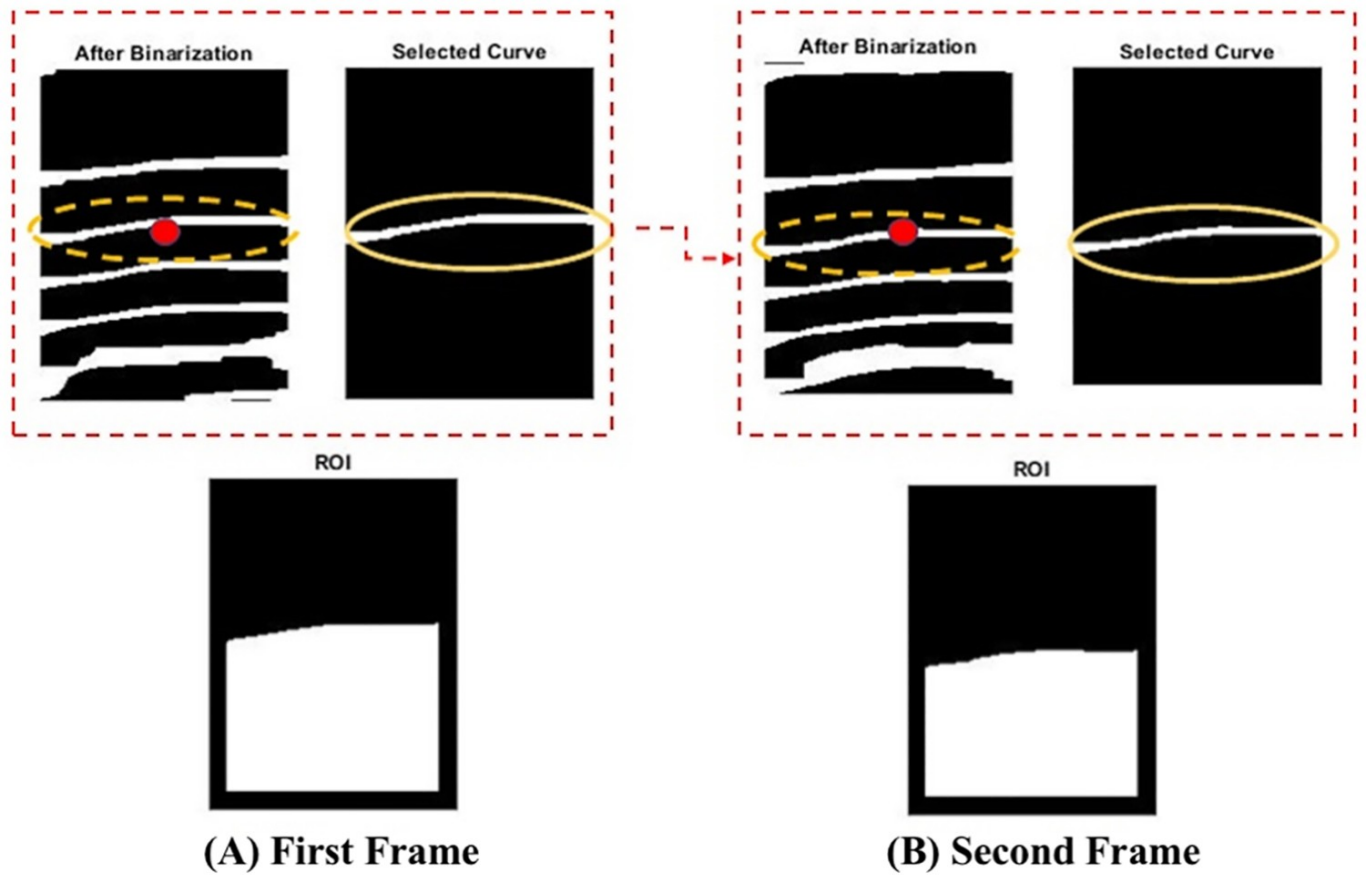
Curve and ROI selection procedures for fingertip movement signal. (A) First frame, and (B) second frame.

The ROI is detected from the binarized image (see Fig 6F). The ROI calculation step is as follows: the curve closest to the center of image (see red dot) is chosen as shown in Fig 7A and 7B, and ROI is the area under the curve which are the white areas in Fig 7A and 7B. The size of the ROI is calculated in each image, and the calculated value is mapped into one point in the fingertip movement signal.

### SQIs calculation step

Both of the color intensity signal and fingertip movement signal are preprocessed by a high pass filter with a cutoff frequency of 0.5 Hz to focus on MNAs in calculating SQIs. The smoothing algorithm is applied to the output of the high pass filtered signal to facilitate the acquisition of the heart rate by getting rid of small fluctuations in the signal. The following SQIs are considered to quantify signal quality in this paper. 1) standard deviation of instantaneous heart rate (*STD*–*HR*), 2) root mean square of the successive differences of peak-to-peak time intervals (*RMSSD*–*T*), and 3) standard deviation of peak values (*STD*–*PV*) [11, 33, 45, 46].

In the SQIs calculation step, the proposed method first divides the signal into multiple segments, calculates SQIs from each segment, and decides whether the segment is corrupted or not. Here, we set the length of segment into 5 seconds, which is determined in a sub-optimal way by grid search algorithm [47]. Since 5 seconds have 150 samples (30 samples/sec for 5 secs), a set of peaks in the *i*^th^ segment consists of the peaks existing in the range of the sample index from (*i* − 1) × 150 to *i* × 150. Here, peaks are defined by a set of local maxima in the signal. A series of peaks are detected in each segment to calculate relevant SQIs which are *STD*–*HR*, *RMSSD*–*T*, and *STD*–*PV*. Denoted by *T*_*i*,*k*_ and *K*_*i*_ the time instant at the *k*^th^ peak of the *i*^th^ segment and the number of peaks in the *i*^*th*^ segment, respectively, each SQI is calculated as follows:

#### Standard deviation of instantaneous heart rate (*STD*–*HR*)

The standard deviation of instantaneous heart rate *STD*–*HR*_*i*_ at the *i*^*th*^ segment is calculated as:

$$\begin{array}{c} {STD\;\;-\;\;HR}_{i}=\sqrt{\frac{1}{K_{i}}\sum_{k=2}^{K}{\left(HR_{i,k}-\bar{HR_{i}}\right)}^{2}} \end{array}$$ (1)

where ${\bar{HR}}_{i}$ is the average value of heart rate at the *i*^*th*^ segment and *HR*_*i*,*k*_ is calculated as:

$$\begin{array}{c} {HR}_{i,k}=\frac{1}{T_{i,k}-T_{i,k-1}}\times 60 \end{array}$$ (2)

Since heart rate remains stable when a subject is stationary, clean segments are expected to have small *STD*–*HR* values. On the other hand, MNA-corrupted segments are expected to have larger *STD*–*HR* values due to irregular peaks caused by MNA.

#### Root mean square of the successive differences of peak-to-peak time intervals (*RMSSD*–*T*)

The root mean square of the successive differences of peak-to-peak time intervals *RMSSD*–*T* at the *i*^th^ segment is calculated as:

$$\begin{array}{c} {RMSSD\;\;-\;\;T}_{i}=\sqrt{\frac{1}{K_{i}-1}\sum_{k=2}^{K_{i}}{\left(\left(T_{i,k+1}-T_{i,k}\right)-\left(T_{i,k}-T_{i,k-1}\right)\right)}^{2}} \end{array}$$ (3)

Since the peaks are irregular in MNA-corrupted segments, while the peaks are regular in clean signals; the *RMSSD*–*T* values of MNA-corrupted segments are expected to be larger compared to those of the clean segments.

#### Standard deviation of peak values (*STD*–*PV*)

The standard deviation of peak values *STD*–*PV* at the *i*^th^ segment is calculated as:

$$\begin{array}{c} {STD\;\;-\;\;PV}_{i}=\sqrt{\frac{1}{K_{i}}\sum_{k=2}^{K}{\left(PV_{i,k}-\bar{PV_{i}}\right)}^{2}} \end{array}$$ (4)

where *PV*_*i*,*k*_ is the *k*^th^ peak value of the *i*^th^ segment and ${\bar{PV}}_{i}$ is the average peak value of the *i*^th^ segment. The *STD*–*PV* values of the MNA-corrupted segments are expected to be larger than those of clean segments since the amplitudes of signals may change due to MNAs while the amplitudes remain stable for the clean ones.

Calculation of the *STD*–*HR*, *RMSSD*–*T*, and *STD*–*PV* requires peak detection as shown in the above equations. Here, a simple peak detection algorithm is applied to the smartphone signals. Specifically, the simple peak detection finds the peaks in the following way: 1) find a series of local maximum points where a local maximum point is defined to be a point having the larger value than its two adjacent neighboring values. 2) Among a series of local maximum points, choose the prominent peaks as output by screening out the local maximum points which have smaller difference compared to the other adjacent local maximum points. The same peak detection algorithm is applied to color intensity and fingertip movement signals.

**Fig 8:**
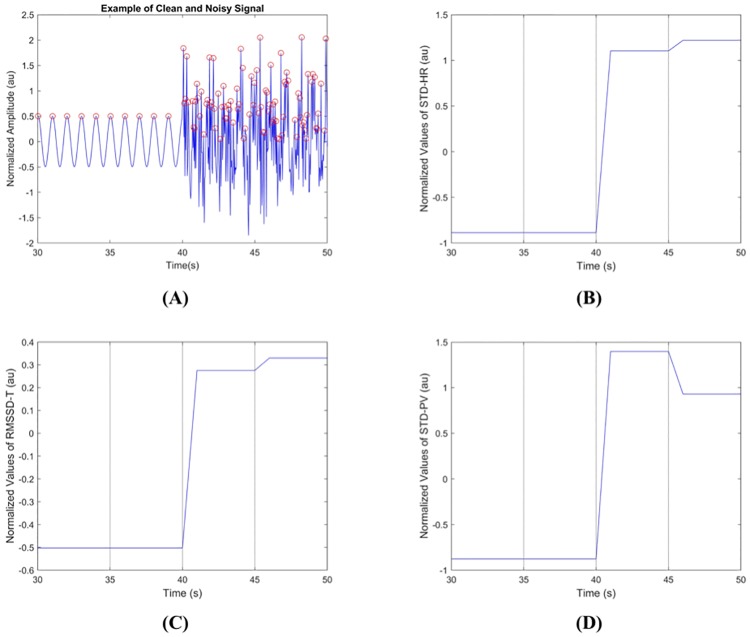
An example of clean and corrupted signals. (A) A sinusoidal signal consisting of clean (30sec—40sec) and corrupted (40sec—50sec) parts, and its corresponding SQIs’ values: (B) standard deviation of instantaneous heart rate (*STD*–*HR*), (C) root mean square of the successive differences of peak-to-peak time intervals (*RMSSD*–*T*), and (D) standard deviation of peak values (*STD*–*PV*).

Fig 8A shows an example of simulated sinusoidal signal without noise (30s-40s) and with noise (40s-50s) period. Here, the noise is added by the simulated additive white Gaussian noise (AWGN) with SNR of -5dB. Fig 8B, 8C and 8D show corresponding SQIs’ values of the signal in Fig 8A. The *STD*–*HR*, *RMSSD*–*T*, and *STD*–*PV* of the corrupted parts are shown to be larger than those of the clean parts since there exist undesired peaks during the noisy period, which makes the SQIs increase compared to the clean period (Fig 8A).

### Proposed diversity method

**Fig 9:**
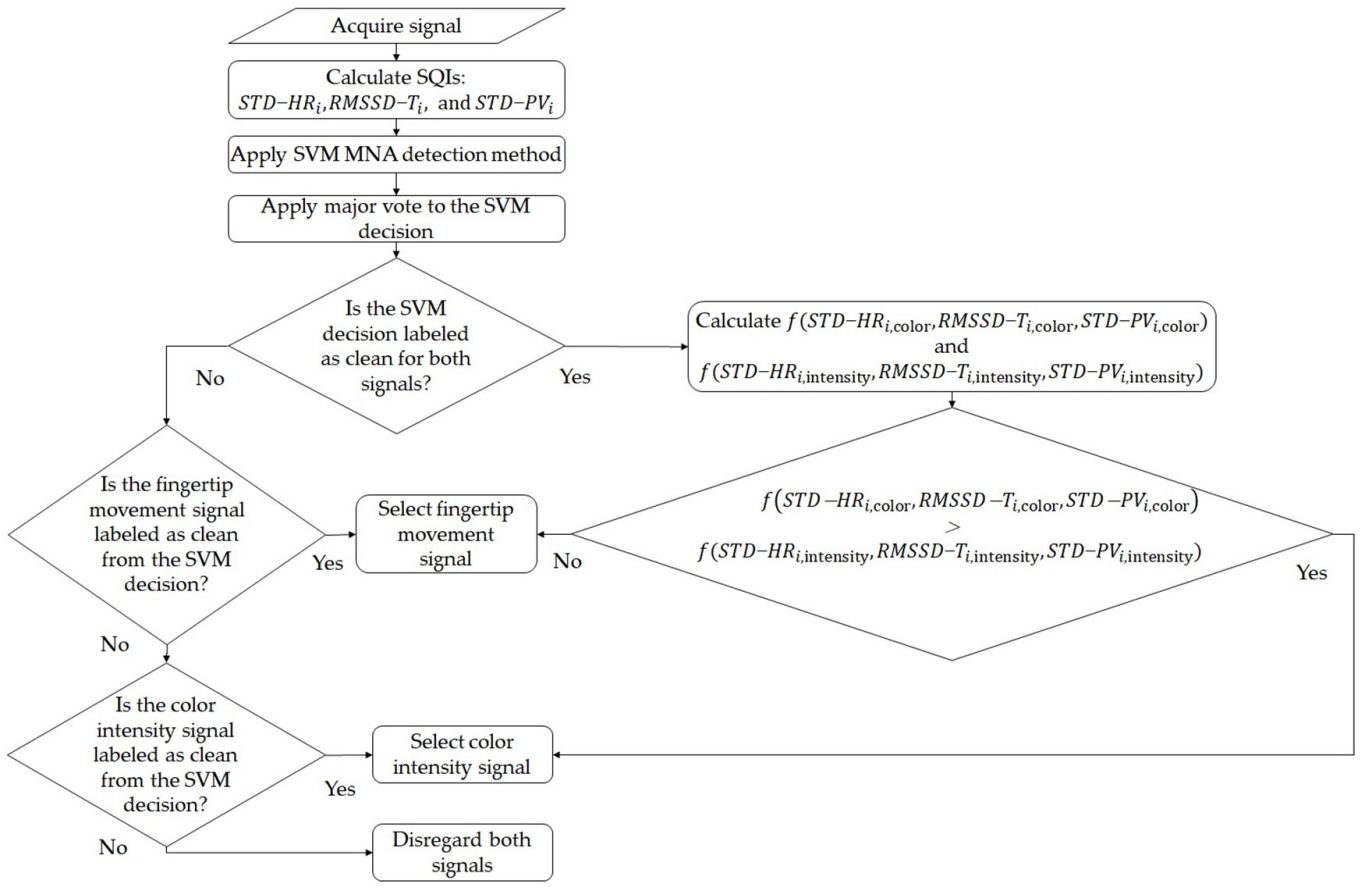
A flow chart of the proposed MNA detection and diversity method.

In the MNA detection and diversity steps, the proposed method performs on each segment, where the segment size is 150 samples (5 seconds). Specifically, the proposed method 1) first detects MNA based on SQIs’ values on each segment, 2) discards the segment if both the color intensity and fingertip movement signals are detected to be corrupted, and 3) selects the better signal based on the signal quality if either of two signals is detected to be clean. We first explain the MNA detection method, and then the signal selection procedure of our diversity method. Fig 9 shows the detailed procedure of our proposed diversity method.

#### MNA decision method

We adopt a concept of support vector machine (SVM) to find the decision boundary between clean and MNA-corrupted segments. Since SVM belongs to supervised machine learning techniques, the decision boundary between different categories is determined not in a predefined way but in an automatic way with a training set. Calculated *STD*–*HR*, *RMSSD*–*T*, and *STD*–*PV* values in each segment are used as input of SVM.

#### Major votes for enhancing MNA detection

We obtain two different SVM boundaries for the color intensity and the fingertip movement signals, separately. The decisions estimated by this SVM model is enhanced by a concept of *major vote*. Specifically, the proposed method applies a concept of major vote method [33] on SVM decision results, i.e., the final MNA decision on a segment is determined by the MNA decision of the neighboring segments as well as the MNA decision of the segment. For example, even though a segment is classified as “clean”, the final decision on the segment is “corrupted” if the neighboring segments are classified as “corrupted”. Specifically, the *i*^th^ segment is clean while its neighboring segments, i.e., (*i* + 1)^th^ and (*i* − 1)^th^ segments, are MNA-corrupted, then the *i*^th^ segment is marked as MNA-corrupted. The authors proposed that this major vote concept needs to be applied right after the MNA decision procedure since if there exist short and intermittent bursts of clean periods during the MNA-corrupted phase, then the inaccurate heart rate information is obtained during this MNA-corrupted phase. This inaccurate heart rate information may raise the hysteresis, and also causes unnecessary computational burden, e.g., calculating the heart rate from that short intermittent segment. On the other hand, corrupted bursts existing between clean segments can give rise to the wrong information if it becomes clean by the major votes. Hence, the major vote algorithm is applied to only the clean bursts existing between corrupted segments.

#### Diversity method

The diversity method chooses between the color intensity and the fingertip movement segments based on these enhanced decision results. The detailed procedure on each *i*^th^ segment is as follows. Here, the evaluation function of SQI parameters is denoted by *f*(⋅), which is the summation value of all the three SQI parameters in each segment. The SQI value of the *i*^th^ segment of the color intensity signal is denoted by *SQI*_*i*,color_, and the SQI value of the *i*^th^ segment of the fingertip movement signal is denoted by *SQI*_*i*,movement_.

1. If both signals are estimated to be corrupted, the segment is rejected.
2. If one signal is estimated to be clean while the other signal is estimated to be corrupted, then the clean signal is selected.
3. If both signals are estimated to be clean, then the signal which has the lower SQI value at the *i*^th^ segment is selected. That is, if *f*(*STD*–*HR*_*i*,color_, *RMSSD*–*T*_*i*,color_, *STD*–*PV*_*i*,color_)<*f*(*STD*–*HR*_*i*,movement_, *RMSSD*–*T*_*i*,movement_, *STD*–*PV*_*i*,movement_), then the color intensity signal is selected. Otherwise, the fingertip movement signal is selected.

## Results

We evaluate the performance of the proposed diversity method for smartphone signals in terms of MNA detection accuracy as well as the ratio of usable (or clean) period. We compare the proposed diversity method to the non-diversity method, i.e., only from the color intensity signal or only from the fingertip movement signal.

### SQI values of clean and corrupted smartphone signals

**Fig 10:**
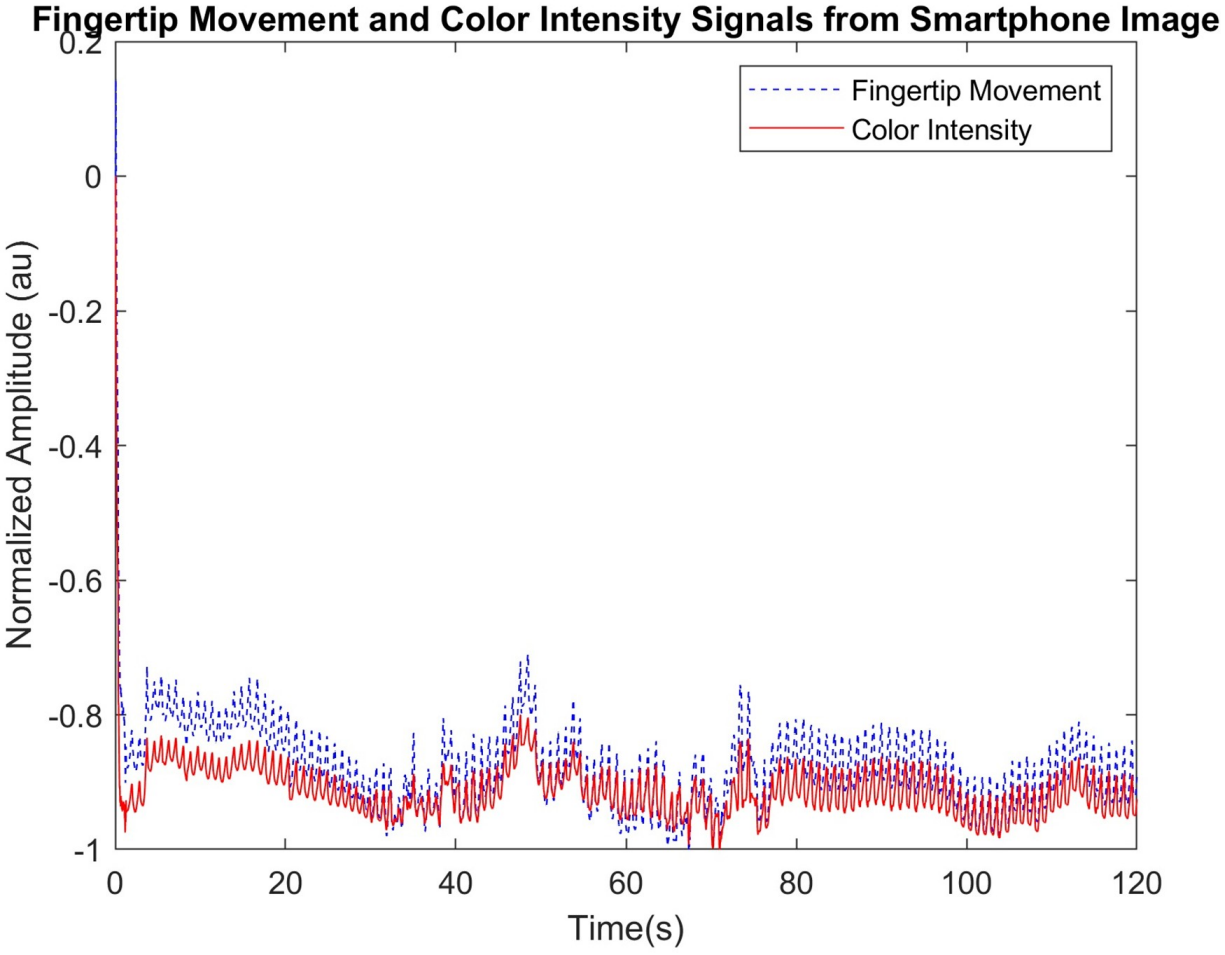
The color intensity and fingertip movement signals acquired using an iPhone.

Fig 10 shows two heterogeneous signals obtained using color intensity and fingertip movement signals from one of our recruited subjects using an iPhone. Here, the color intensity signal is relatively less corrupted by MNA compared to the fingertip movement signal. Specifically, fingertip movement signal is corrupted by MNA during 5-15 seconds, 20-25 seconds, 30-65 second, and 80-85 seconds while the other part is clean. On the other hand, the average intensity PPG is corrupted by MNA only during 30-65 seconds while the other part is clean.

**Fig 11:**
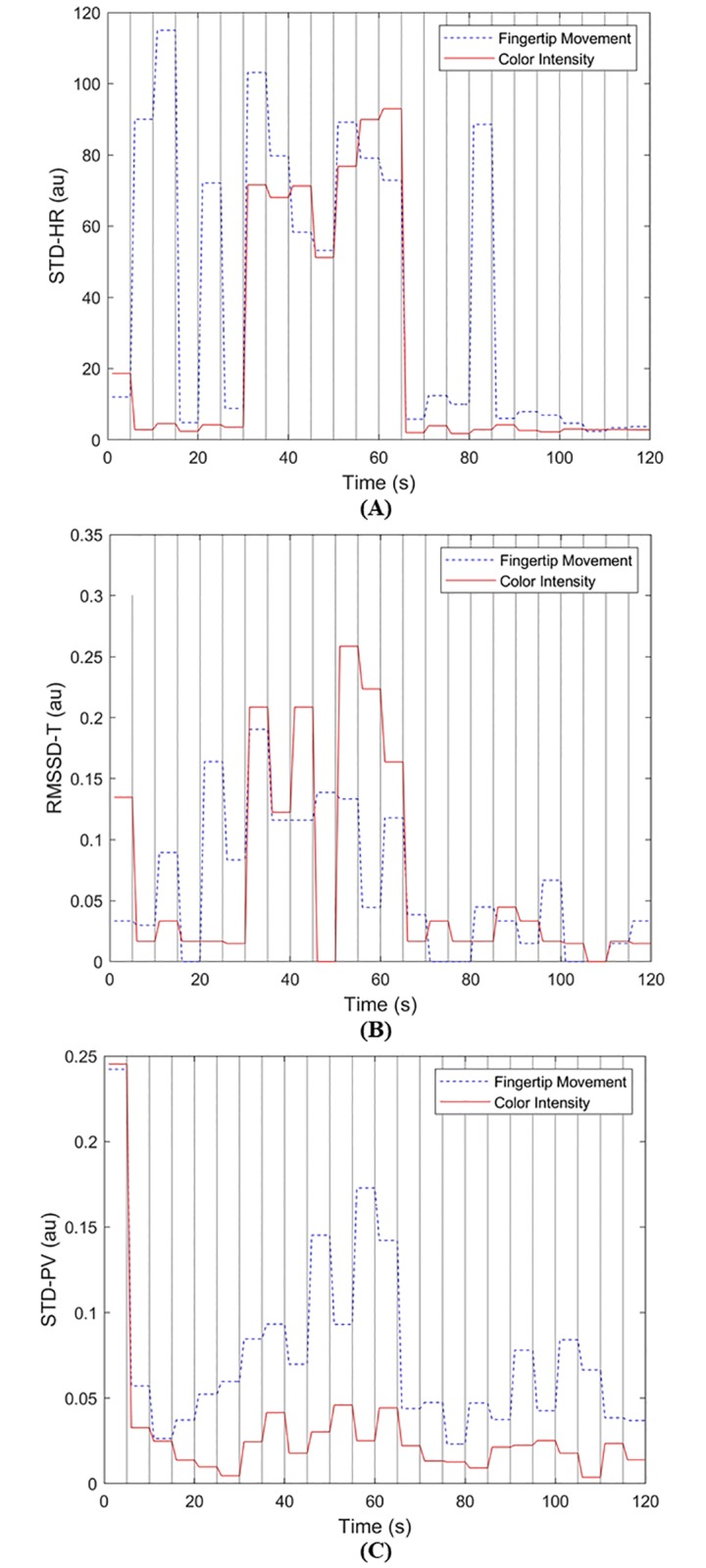
The SQIs’ values acquired from heterogeneous color intensity and fingertip movement signals shown in Fig 10. (A) Standard deviation of instantaneous heart rate (*STD*–*HR*), (B) root mean square of the successive differences of peak-to-peak time intervals (*RMSSD*–*T*), and (C) standard deviation of peak values (*STD*–*PV*).

**Fig 12:**
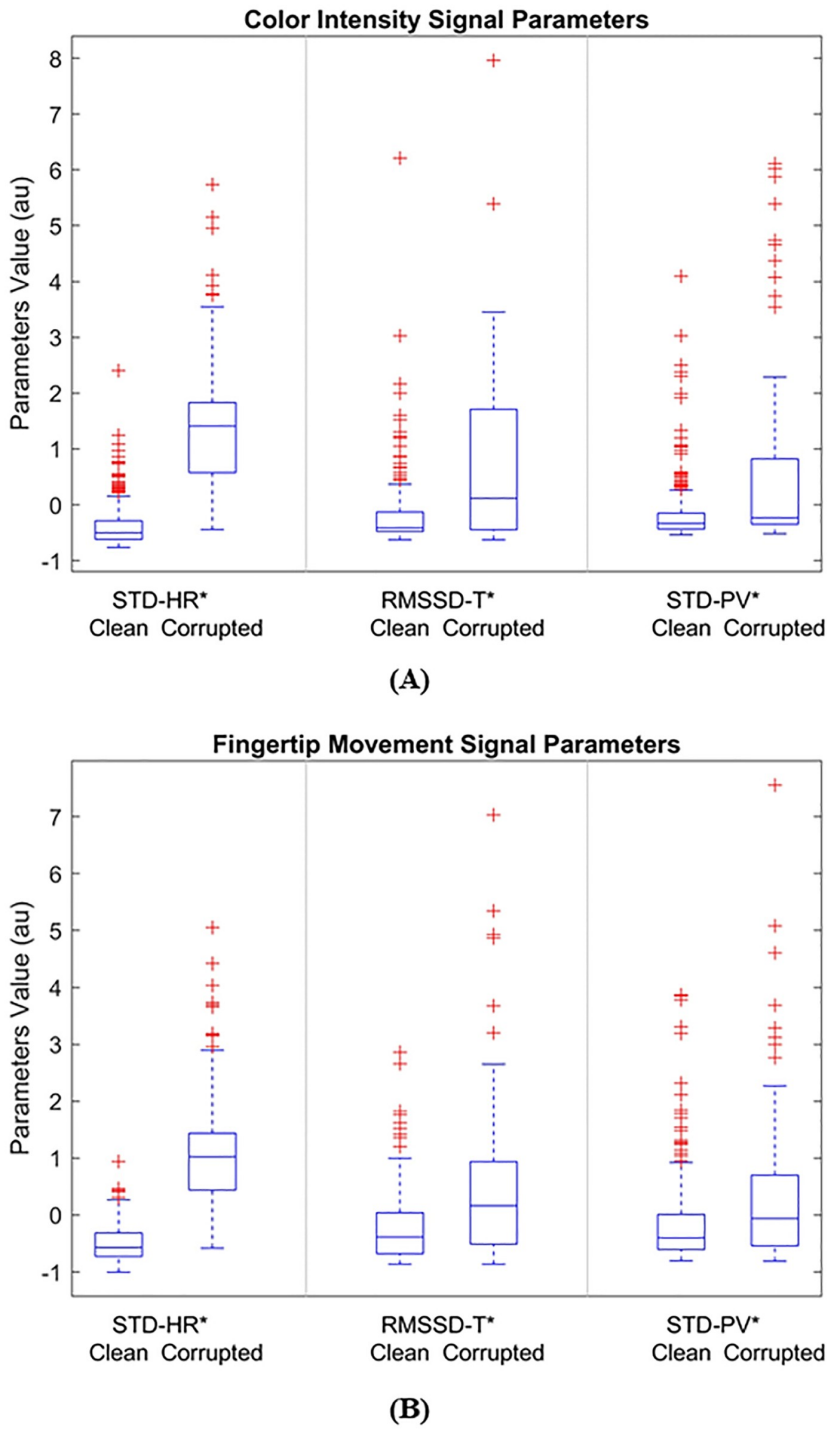
Box plots for mean of SQIs of clean and corrupted segments. (A) color intensity and (B) fingertip movement PPG signals. Central mark in each of the plot denotes median value of the SQIs while edges of the box denote 25^th^ and 75^th^ percentile value. The bars above and below to the box represent the extreme data points excluded from being considered outliers, and points represented by ‘+’ are considered outliers.

Fig 11 shows the SQIs’ values for the color intensity and the fingertip movement signals in Fig 10. Fig 12 shows the distribution of the three SQIs’ values using box plots. The average SQIs’ values of corrupted segments are shown to be larger than (or different from) those of clean parts as shown in Fig 11.

The *p*-values between SQIs’ values of clean and corrupted segments are presented in Table 1. The results show that for all three SQIs the *p*-values are less than 0.05 (*p* < 0.05). This indicates that the SQI parameters of both signals are significantly different between the clean and corrupted segments.

**Table 1:** The *p*-values between SQIs’ values of clean and corrupted segments.

	STR-HR	RMSSD-T	STD-PV
Color Intensity	3.25e-70	6.16e-11	1.75e-4
Fingertip Movement	1.02e-67	6.73e-15	1.66e-10

### Performance evaluation of MNA detection and diversity effect

To validate the accuracy of the proposed SQIs in discriminating whether the segment is usable or not, we have the following annotation procedure. Annotations are performed segment-by-segment. Denoted by *avgHR*_*i*,mea_ and *avgHR*_*i*,ref_ the average heart rate values for the *i*^th^ segment of smartphone and NeXus 10 mark-II signals, respectively, the *i*^th^ segment of the smartphone signal is annotated as corrupted if |*avgHR*_*i*,mea_ − *avgHR*_*i*,ref_| ≥ *TH*_diffHR_. Otherwise, the *i*^th^ segment of the smartphone signal is annotated as clean. Here the *TH*_diffHR_ is defined as 8 beats per minute (bpm). This threshold is defined based on the sampling rate of the smartphone and the NeXus device. The sampling rate of the smartphone is 30Hz while the sampling rate of the NeXus device is 32Hz. The maximum value of normal heart rate was considered as 120bpm in which one sample error in the signal would cause changes of 8bpm in the heart rate.

We adopt support vector machine (SVM) to get a decision boundary between clean and MNA classes. For the fingertip movement signal, the total number of segments is 359 among which the numbers of clean and corrupted segments are 255 and 104, respectively. For the color intensity signal, on the other hand, the total number of segments is 359 which consists of 286 clean segments and 73 corrupted segments. The ratio between the clean and corrupted segments is 70% to 30% for fingertip movement signal while the ratio is 79% to 21% for color intensity signal. The 5-fold validation is adopted in training and testing stages. During the training stage, SQIs’ values of clean and corrupted segments are used as input training data and the corresponding annotations are used as labels for the input training data. During the test stage, the SQIs’ values of the unknown segments are used as input test data, and the accuracy is calculated using the following equation by comparing the proposed SQI’s estimation on the segment by the SVM to the corresponding annotations:

$$\begin{array}{c} accuracy=\frac{N_{\text{tp}}+N_{\text{tn}}}{N_{\text{tp}}+N_{\text{tn}}+N_{\text{fp}}+N_{\text{fn}}} \end{array}$$ (5)

where, *N*_tp_, *N*_tn_, *N*_fp_, and *N*_fn_ are the number of true positive, true negative, false positive and false negative segments, respectively.

On the other hand, the proposed diversity detection method is evaluated in terms of usable period ratio which is defined as:

$$\begin{array}{c} usable\;period\;ratio=\frac{usable\;period}{total\;duration} \end{array}$$ (6)

We evaluate the performance of the proposed diversity method by comparing *usable period ratio* of the proposed diversity method to that of the non-diversity methods.

#### MNA detection

To evaluate the MNA detection performance, our collected smartphone signals are segmented and annotated by NeXus 10 mark-II signals. Four types of annotations are assigned to each segment: 1) Red (R, color intensity signal is clean but fingertip movement signal is corrupted), 2) Blue (B, fingertip movement signal is clean but color intensity signal is corrupted), 3) Green (G=R∪B either of signals is clean), and 4) Black (BL, both signals are corrupted). Here, G and BL are used for the reference while R, B, and BL are used for the decisions made by the SVM.

Specifically, it is *True Positive* if the reference is G (= R∪B) and the decision made by the SVM is R or B (= R∪B). On the other hand, it is *True Negative* if the annotation given to the time segment is BL and the output of our proposed method is found to be BL. It is *False Positive* if at least one of the signals were annotated as G (= R∪B) while the method estimates it to be BL. Finally, it is False Negative if both of the signals are annotated to be BL while the method estimates it to be either R or B.

**Fig 13:**
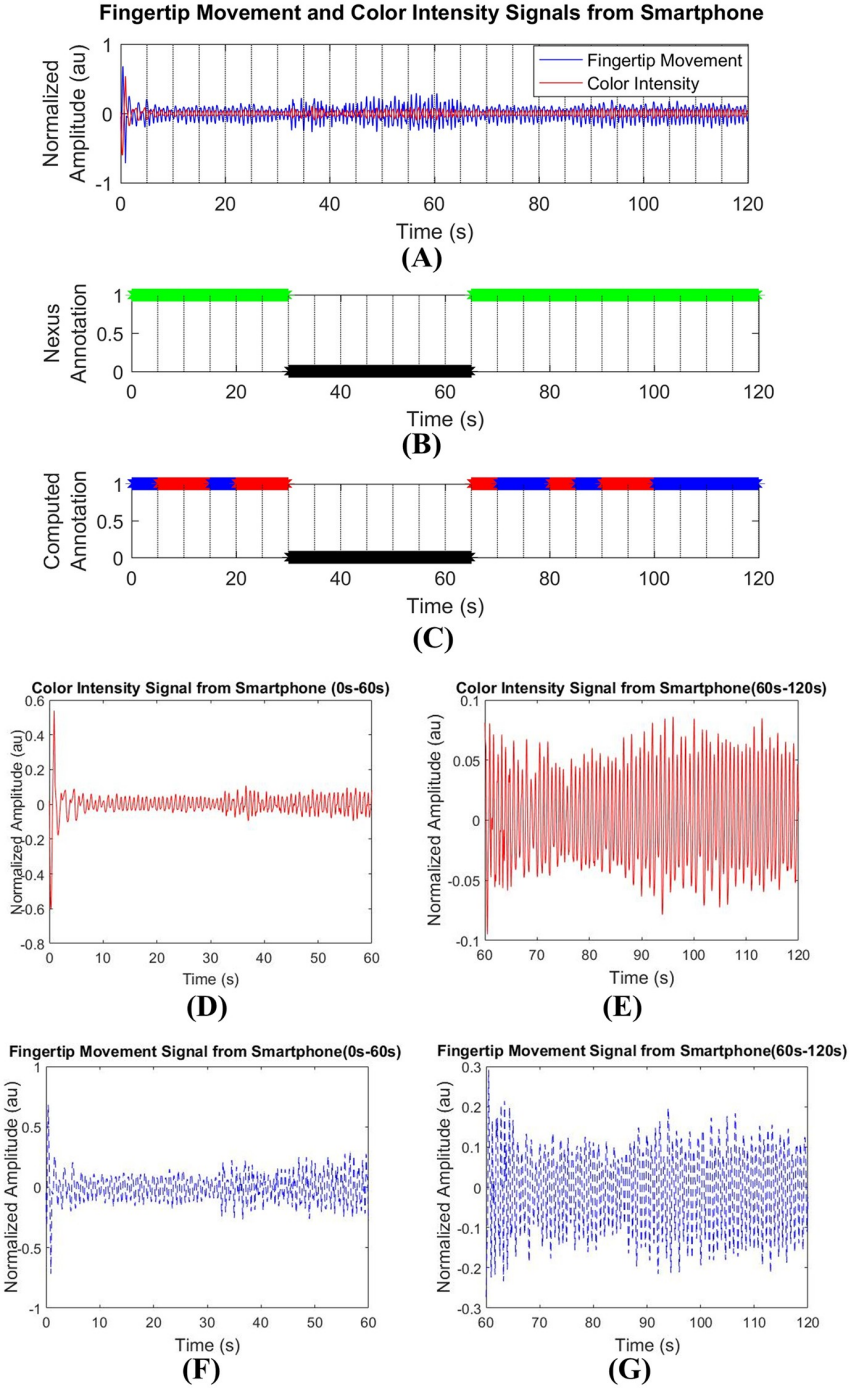
MNA detection decisions made by our proposed MNA detection method. (A) filtered fingertip movement and color intensity signals, (B) annotation from NeXus signal, (C) estimation of our proposed diversity method, (D) first-half of the color intensity signal (0s-60s), (E)second-half of the color intensity signal (60s-120s), (F) first-half of the fingertip movement signal (0s-60s), and (G) second-half of the fingertip movement signal (60s-120s).

Fig 13A shows the color intensity and the fingertip movement signals acquired from a single smartphone video recording. Fig 13B shows the decision about clean/noisy parts of the signals made by annotation procedure with the NeXus 10 mark-II signals while Fig 13C shows estimation results of our proposed MNA detection method. Comparing Fig 13B with Fig 13C, the proposed MNA detection method is shown to give highly accurate estimation.

Table 2 shows the accuracy of our proposed method. The proposed method shows MNA detection accuracy of 93.0% for the color intensity and 93.3% for the fingertip movement.

**Table 2:** Accuracy of the MNA detection using the proposed method.

	Color Intensity	Fingertip Movement
Proposed Method	93.0%	93.3%

#### Diversity effect

Usable period ratio (or clean period ratio) of our proposed diversity method is compared to those of the color intensity and the fingertip movement signals in Table 3. The usable period ratio of the proposed diversity method is shown to be 85.23% while those of the color intensity and fingertip movement signals are shown to be 80.22% and 71.30%, respectively. This result shows that our proposed diversity method increases the portion of usable period by 6.25% and 19.53% compared to the color intensity only and the fingertip movement only signals, respectively.

**Table 3:** Usable period ratio (clean segments ratio) using the proposed method.

	Diversity	Color Intensity	Fingertip Movement
Ratio of Usable Clean Segments to Total Segments	85.23%	80.22%	71.30%

## Discussion

In this paper, a diversity method for two heart rhythm signals,—which are respectively obtained by assessing the color intensity and the fingertip movement signals from a single smartphone camera recording- is proposed to reliably and continuously get heart rhythm information in the presence of MNAs. To achieve this, our proposed diversity method 1) acquires two different types of smartphone signals, 2) quantifies the respective amount of MNAs in two heterogeneous signals based on the proposed SQIs’ values on a segment basis, and finally 3) exploits diversity from the MNA detection results of two signals on a segment basis.

One of the advantages of the proposed method is in the signal acquisition step. That is, the computational complexity is not increased in getting two heterogeneous signals since it is obtained from a single smartphone recording. Hence, it does not require additional signal acquisition procedure. The other advantage of the proposed method comes from the diversity gain in the usable period ratio (or clean period ratio), which is compared to the conventional method, i.e. the color intensity or the fingertip movement signal.

We have evaluated our proposed method by applying it to both MNA-free and MNA-corrupted smartphone signals acquired from 15 healthy subjects. The experimental results have shown that the proposed SQIs’ values are significantly different between MNA-free and MNA-corrupted signals. Specifically, the paired t-test was performed to determine whether there is significant difference (*p* < 0.05 at 95% confidence interval) between the SQIs’ values signal obtained from MNA-free signals and MNA-corrupted signals. Especially, we adopted SVM to set the boundary classifying MNA-clean and MNA-corrupted segments. As input of the SVM, three SQIs are considered: 1) standard deviation of instantaneous heart rate (*STD*–*HR*), 2) root mean square of the successive differences of peak-to-peak time intervals (*RMSSD*–*T*), and 3) standard deviation of peak values (*STD*–*PV*). We compared the MNA detection performance in our proposed method to the other MNA detection techniques [48, 49] which used *RMSSD*–*T* parameter only and *STD*–*PV* parameter only to detect MNA. Table 4 shows the MNA detection accuracies of the proposed method, the RMSSD-T only [48], and the STD-PV only method [48, 49]. As shown in Table 4, the accuracy of the proposed method is around 93% for both the color intensity and the fingertip movement signals while the RMSSD-T only method in [48] gives 78% and 70% accuracies for the color intensity and the fingertip movement signals, respectively. The accuracy for the STD-PV only method [48, 49] is 81.9% and 71.6% for the color intensity and the fingertip movement signals, respectively. As a result, our method performs better MNA detection than RMSSD-T only or STD-PV only methods in [48, 49] do.

**Table 4:** Comparison of MNA detection performance between the proposed method, the RMSSD-T only method in [48], and the STD-PV only method in [48, 49].

	Color Intensity	Fingertip Movement
Proposed Method (STD-HR, RMSSD-T and STD-PV)	93.0%	93.3%
RMSSD-T Only Method [48]	78.0%	70.0%
STD-PV Only Method [48]	81.9%	71.6%

The experimental results also have shown that our proposed diversity method with these MNA detection results provides 6.25% and 19.53% higher usable clean periods compared to the conventional color intensity-only or fingertip movement-only signals. The proposed method in this paper is expected to be useful for getting continuous physiological information using different types of or multiple signals from a smartphone, including heart rhythm information, in the presence of motions or noise artifacts.

**Fig 14:**
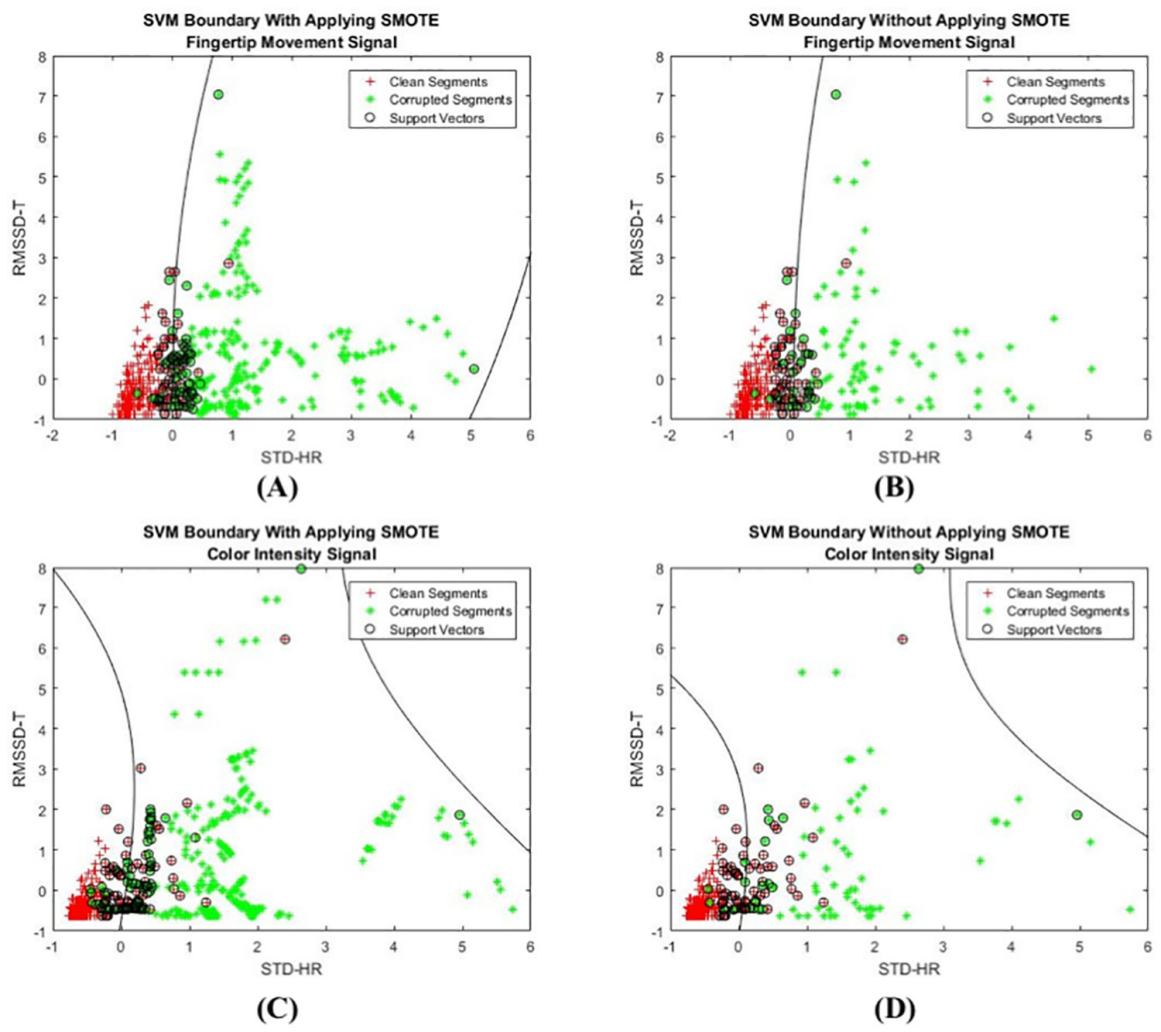
The two-dimensional representation of the SVM decision boundary for fingertip movement and color intensity signals. The green stars are corrupted samples and red crosses represent the clean samples. The samples selected as support vectors are marked with black circles. (A) The SVM boundary after applying the SMOTE technique on fingertip movement signal, (B) the SVM boundary of fingertip movement signal without the SMOTE technique, (C) the SVM boundary after applying the SMOTE technique on color intensity signal, and (D) the SVM boundary of color intensity signal without the SMOTE technique.

Especially, we adopted the SVM to set the boundary classifying MNA-clean and MNA-corrupted segments. In the SVM classifier, the ratio between the clean and corrupted segments were 70% to 30% for fingertip movement signal and 79% to 21% for color intensity signal. To study the effect of imbalanced data, we adopted the synthetic minority over-sampling technique (SMOTE) [50] to increase the number of corrupted samples (minority class) and make it the same size as the number of clean segments. The SMOTE technique creates synthetic data by using *n* number of nearest neighbors of the features. This technique maps a sample data with the dimension of (*S*,*f*) to the new data with the dimension of (*S*’,*f*) where *S* is the original sample size of data, *S*’ is the size of oversampled data and *f* is the size of the feature vectors. First, *n* nearest neighbors of a sample are selected randomly. The difference of the feature vector of the sample with the *n* nearest neighbors are derived. These feature vectors are multiplied with a random value between 0 and 1 to create the final oversampled samples [50]. The accuracy of the MNA detection method after adopting the SMOTE approach is 90.0% for color intensity and 92.5% for fingertip movement. These values are 93.0% for color intensity and 93.3% for fingertip movement without applying the SMOTE. The difference between the accuracy from the SMOTE approach and without the SMOTE approach is observed to be less than 5%. Fig 14 shows the SVM decision boundary for both fingertip movement and color intensity signals before and after adopting the SMOTE technique. Fig 14A is two-dimensional representation of the SVM boundary and support vectors when the SMOTE technique is applied. On the other hand, two-dimensional representation of the SVM boundary without the SMOTE is shown in Fig 14B. The SVM boundary decision for the color intensity signal after and before applying the SMOTE technique is shown in Fig 14C and 14D. As shown in Fig 14A and 14C, by adopting the SMOTE technique the number of corrupted segments (green star) is increased compared to those in Fig 14B and 14D while the number of clean segments remains the same. However, after adopting the SVM, there is not too much difference between the selected samples as support vectors in both cases as shown in Fig 14. Moreover, the main boundary line positions have not changed after applying the SMOTE. The results indicate that SVM is robust to the imbalanced data in this example.

Moreover, we performed the diversity method on the results of the MNA detection method with the SMOTE approach. The ratio of usable segments after applying the SMOTE technique is 72.71% and without the SMOTE technique is 80.22% for the color intensity. For the fingertip movement signal, the ratio of the usable segments is 67.96% and 71.30% without the SMOTE. Therefore, the ratio of usable segments is higher for each type of signals without the SMOTE technique. With the diversity approach, the enhanced decision results enable us to select between the color intensity and the fingertip movement signals based on the quality of the signal (clean or corrupted). As a result, the ratio of usable clean segments is 81.89% after adopting the SMOTE technique while it is 85.23% without the SMOTE. The results of the diversity method provides12.6% and 20.5% increase in the ratio of usable clean periods compared to the conventional color intensity-only or fingertip movement-only signals after adopting the SMOTE. Although the values of increment are slightly higher compared to the increment ratio when the SMOTE is not applied (6.25% for color intensity and 19.53% for fingertip movement signal), the ratio of usable clean segment is less after applying the SMOTE for each of the color intensity, fingertip movement and diversity method.

## References

[1] McManus DD, Chong JW, Soni A, Saczynski JS, Esa N, Napolitano C, et al PULSE-SMART: Pulse-Based Arrhythmia Discrimination Using a Novel Smartphone Application. Journal of Cardiovascular Electrophysiology. 2016;27(1):51–7. 10.1111/jce.12842 26391728 PMC4768310

[2] Paiva S. Mobile Solutions and Their Usefulness in Everyday Life: Springer; 2019.

[3] (WHO) WHO. Cardiovascular disease. 2018. Available from: https://www.who.int/cardiovascular_diseases/en/.

[4] Chung H, Ko H, Thap T, Jeong C, Noh S-E, Yoon K-H, et al Smartphone Based Cardiac Rehabilitation Program: Feasibility Study. PLOS ONE. 2016;11(8):e0161268 10.1371/journal.pone.0161268 27551969 PMC4995057

[5] Mortelmans C. Validation of a new smartphone application (“FibriCheck”) for the diagnosis of atrial fibrillation in primary care. Leuven; 2016.

[6] Chan PH, Wong CK, Poh Yukkee C, Pun L, Leung Wangie WC, Wong YF, et al Diagnostic Performance of a Smartphone Based Photoplethysmographic Application for Atrial Fibrillation Screening in a Primary Care Setting. Journal of the American Heart Association. 2016;5(7):e003428 10.1161/JAHA.116.003428 27444506 PMC5015379

[7] Chong JW, Esa N, McManus DD, Chon KH. Arrhythmia Discrimination Using a Smart Phone. IEEE Journal of Biomedical and Health Informatics. 2015;19(3):815–24. 10.1109/JBHI.2015.2418195 25838530 PMC6599713

[8] Shelley KH. Photoplethysmography: beyond the calculation of arterial oxygen saturation and heart rate. Anesthesia and analgesia. 2007;105(6 Suppl):S31–6. 10.1213/01.ane.0000269512.82836.c9 18048895

[9] Hertzman AB. Observations on the finger volume pulse recorded photoelectrically. Am J Physiol. 1937;119:334–5.

[10] Foster MDAD, Neumann MDC, Rovenstine MDEA. PERIPHERAL CIRCULATION DURING ANESTHESIA, SHOCK AND HEMORRHAGE; THE DIGITAL PLETHYSMOGRAPH AS A CLINICAL GUIDE. Anesthesiology. 1945;6(3):246–57. 10.1097/00000542-194505000-00004

[11] Chong JW, Cho CH, Tabei F, Le-Anh D, Esa N, McManus DD, et al Motion and Noise Artifact-Resilient Atrial Fibrillation Detection using a Smartphone. IEEE Journal on Emerging and Selected Topics in Circuits and Systems. 2018 10.1109/JETCAS.2018.2818185 30687580 PMC6345530

[12] Zaman R, Cho C, Hartmann-Vaccarezza K, Phan T, Yoon G, Chong J. Novel Fingertip Image-Based Heart Rate Detection Methods for a Smartphone. Sensors. 2017;17(2):358 10.3390/s17020358 PMC5336072 28208678

[13] Bolkhovsky JB, Scully CG, Chon KH, editors. Statistical analysis of heart rate and heart rate variability monitoring through the use of smart phone cameras. 2012 Annual International Conference of the IEEE Engineering in Medicine and Biology Society; 2012 Aug. 28—2012 Sept. 1 2012. 10.1109/EMBC.2012.6346253 23366214

[14] Garcia-Agundez A, Dutz T, Goebel S. Adapting smartphone-based photoplethysmograpy to suboptimal scenarios. Physiological measurement. 2017;38(2):219–32. 10.1088/1361-6579/aa51db 28099163

[15] Li KHC, White FA, Tipoe T, Liu T, Wong MCS, Jesuthasan A, et al The Current State of Mobile Phone Apps for Monitoring Heart Rate, Heart Rate Variability, and Atrial Fibrillation: Narrative Review. JMIR Mhealth Uhealth. 2019;7(2):e11606 10.2196/11606 30767904 PMC6396075

[16] Li K, Warren S. A Wireless Reflectance Pulse Oximeter With Digital Baseline Control for Unfiltered Photoplethysmograms. IEEE Transactions on Biomedical Circuits and Systems. 2012;6(3):269–78. 10.1109/TBCAS.2011.2167717 23853148

[17] Li K, Warren S, Natarajan B. Onboard Tagging for Real-Time Quality Assessment of Photoplethysmograms Acquired by a Wireless Reflectance Pulse Oximeter. IEEE Transactions on Biomedical Circuits and Systems. 2012;6(1):54–63. 10.1109/TBCAS.2011.2157822 23852745

[18] Yan Y-s, Poon CCY, Zhang Y-t. Reduction of motion artifact in pulse oximetry by smoothed pseudo Wigner-Ville distribution. Journal of NeuroEngineering and Rehabilitation. 2005;2:3 10.1186/1743-0003-2-3 15737241 PMC553999

[19] Rolando Hong E, Miguel Sautié C, Jersys Falcón R, José Luis Hernández C. Analysis of the photoplethysmographic signal by means of the decomposition in principal components. Physiological measurement. 2002;23(3):N17 10.1088/0967-3334/23/3/402 12214766

[20] Kim BS, Yoo SK. Motion artifact reduction in photoplethysmography using independent component analysis. IEEE Transactions on Biomedical Engineering. 2006;53(3):566–8. 10.1109/TBME.2005.869784 16532785

[21] Lee J, Jung W, Kang I, Kim Y, Lee G. Design of filter to reject motion artifact of pulse oximetry. Computer Standards & Interfaces. 2004;26(3):241–9. 10.1016/S0920-5489(03)00077-1

[22] Peng F, Zhang Z, Gou X, Liu H, Wang W. Motion artifact removal from photoplethysmographic signals by combining temporally constrained independent component analysis and adaptive filter. BioMedical Engineering OnLine. 2014;13:50 10.1186/1475-925X-13-50 24761769 PMC4021027

[23] Ram MR, Madhav KV, Krishna EH, Komalla NR, Reddy KA. A Novel Approach for Motion Artifact Reduction in PPG Signals Based on AS-LMS Adaptive Filter. IEEE Transactions on Instrumentation and Measurement. 2012;61(5):1445–57. 10.1109/TIM.2011.2175832

[24] Ram MR, Madhav KV, Krishna EH, Reddy KN, Reddy KA, editors. Use of spectral estimation methods for computation of SpO2 from artifact reduced PPG signals. 2011 IEEE Recent Advances in Intelligent Computational Systems; 2011 22-24 Sept. 2011.

[25] Rusch TL, Sankar R, Scharf JE. Signal processing methods for pulse oximetry. Computers in Biology and Medicine. 1996;26(2):143–59. 10.1016/0010-4825(95)00049-6 8904288

[26] Krishnan R, Natarajan B, Warren S. Two-Stage Approach for Detection and Reduction of Motion Artifacts in Photoplethysmographic Data. IEEE Transactions on Biomedical Engineering. 2010;57(8):1867–76. 10.1109/TBME.2009.2039568 20172800

[27] Selvaraj N, Mendelson Y, Shelley KH, Silverman DG, Chon KH, editors. Statistical approach for the detection of motion/noise artifacts in Photoplethysmogram. 2011 Annual International Conference of the IEEE Engineering in Medicine and Biology Society; 2011 Aug. 30 -2011 Sept. 3 2011. 10.1109/IEMBS.2011.6091232 22255454

[28] Foo JYA, Wilson SJ. A computational system to optimise noise rejection in photoplethysmography signals during motion or poor perfusion states. Medical and Biological Engineering and Computing. 2006;44(1):140–5. 10.1007/s11517-005-0008-y 16929932

[29] Dash S, Chon KH, Lu S, Raeder EA. Automatic Real Time Detection of Atrial Fibrillation. Annals of Biomedical Engineering. 2009;37(9):1701–9. 10.1007/s10439-009-9740-z 19533358

[30] Karlen W, Kobayashi K, Ansermino JM, Dumont GA. Photoplethysmogram signal quality estimation using repeated Gaussian filters and cross-correlation. Physiological measurement. 2012;33(10):1617 10.1088/0967-3334/33/10/1617 22986287

[31] Boreom L, Jonghee H, Hyun Jae B, Jae Hyuk S, Kwang Suk P, Won Jin Y. Improved elimination of motion artifacts from a photoplethysmographic signal using a Kalman smoother with simultaneous accelerometry. Physiological measurement. 2010;31(12):1585 10.1088/0967-3334/31/12/003 20980715

[32] Yu C, Liu Z, McKenna T, Reisner AT, Reifman J. A Method for Automatic Identification of Reliable Heart Rates Calculated from ECG and PPG Waveforms. Journal of the American Medical Informatics Association: JAMIA. 2006;13(3):309–20. 10.1197/jamia.M1925 16501184 PMC1513657

[33] Chong JW, Dao DK, Salehizadeh SMA, McManus DD, Darling CE, Chon KH, et al Photoplethysmograph Signal Reconstruction Based on a Novel Hybrid Motion Artifact Detection–Reduction Approach. Part I: Motion and Noise Artifact Detection. Annals of Biomedical Engineering. 2014;42(11):2238–50. 10.1007/s10439-014-1080-y 25092422

[34] Nakamura M, Nakamura J, Lopez G, Shuzo M, Yamada I. Collaborative processing of wearable and ambient sensor system for blood pressure monitoring. Sensors. 2011;11(7):6760–70. 10.3390/s110706760 22163984 PMC3231663

[35] Rajive J. Multisensor fusion: a minimal representation framework: World Scientific; 1999.

[36] Mason C, Tarassenko L, editors. Quantitative assessment of respiratory derivation algorithms. 2001 Conference Proceedings of the 23rd Annual International Conference of the IEEE Engineering in Medicine and Biology Society; 2001: IEEE.

[37] Nemati S, Malhotra A, Clifford GD. Data fusion for improved respiration rate estimation. EURASIP journal on advances in signal processing. 2010;2010:10 10.1155/2010/926305 PMC2929127 20806056

[38] Li Q, Clifford GD. Signal quality and data fusion for false alarm reduction in the intensive care unit. Journal of electrocardiology. 2012;45(6):596–603. 10.1016/j.jelectrocard.2012.07.015 22960167

[39] Orphanidou C, Bonnici T, Charlton P, Clifton D, Vallance D, Tarassenko L. Signal-quality indices for the electrocardiogram and photoplethysmogram: Derivation and applications to wireless monitoring. IEEE journal of biomedical and health informatics. 2015;19(3):832–8. 10.1109/JBHI.2014.2338351 25069129

[40] Pimentel MA, Santos MD, Springer DB, Clifford GD. Heart beat detection in multimodal physiological data using a hidden semi-Markov model and signal quality indices. Physiological measurement. 2015;36(8):1717 10.1088/0967-3334/36/8/1717 26218536

[41] NeXus by Mind Media. 2018. Available from: https://www.mindmedia.com/en/products/nexus-10-mkii/.

[42] Peng R-C, Zhou X-L, Lin W-H, Zhang Y-T. Extraction of Heart Rate Variability from Smartphone Photoplethysmograms. Computational and Mathematical Methods in Medicine. 2015;2015:11 10.1155/2015/516826 PMC4309304 25685174

[43] Tschumperlé D, Deriche R. Anisotropic diffusion partial differential equations for multichannel image regularization: Framework and applications. Advances in Imaging and Electron Physics. 2007;145:149–209. 10.1016/S1076-5670(06)45004-7

[44] Perona P, Malik J. Scale-space and edge detection using anisotropic diffusion. IEEE Transactions on pattern analysis and machine intelligence. 1990;12(7):629–39. 10.1109/34.56205

[45] Couceiro R, Carvalho P, Paiva RP, Henriques J, Muehlsteff J, editors. Detection of motion artifacts in photoplethysmographic signals based on time and period domain analysis. 2012 Annual International Conference of the IEEE Engineering in Medicine and Biology Society; 2012 Aug. 28 -2012 Sept. 1. 2012. 10.1109/EMBC.2012.6346497 23366458

[46] Tabei F, Kumar R, Phan TN, McManus DD, Chong JW. A Novel Personalized Motion and Noise Artifact (MNA) Detection Method for Smartphone Photoplethysmograph (PPG) Signals. IEEE Access. 2018;6:60498–512. 10.1109/ACCESS.2018.2875873 PMC6602087 31263653

[47] Hsu C-W, Chang C-C, Lin C-J. A practical guide to support vector classification. 2003.

[48] Manis G, Alexandridi A, Nikolopoulos S, Davos K, editors. The effect of white noise and false peak Detection on HRV Analysis. International Workshop on Biosignal Processing and Classification; 2005.

[49] Friesen GM, Jannett TC, Jadallah MA, Yates SL, Quint SR, Nagle HT. A comparison of the noise sensitivity of nine QRS detection algorithms. IEEE Transactions on Biomedical Engineering. 1990;37(1):85–98. 10.1109/10.43620 2303275

[50] Chawla NV, Bowyer KW, Hall LO, Kegelmeyer WP. SMOTE: synthetic minority over-sampling technique. Journal of artificial intelligence research. 2002;16:321–57. 10.1613/jair.953

